# Developing an open educational resource for open research: Protocol for the PaPOR TRAIL project

**DOI:** 10.12688/hrbopenres.13171.1

**Published:** 2020-11-12

**Authors:** Sophia Egan, Mary Tobin, Brendan Palmer, Aoife Coffey, Darren Dahly, Catherine Houghton, Eoghan Ó Carragáin, Elaine Toomey, Samantha Dockray, Karen Matvienko-Sikar

**Affiliations:** 1 School of Public Health, University College Cork, Cork, Ireland; 2 Health Research Board Clinical Research Facility Cork, University College Cork, Cork, Ireland; 3 UCC Library, University College Cork, Cork, Ireland; 4 School of Nursing and Midwifery, National University of Ireland, Galway, Galway, Ireland; 5 School of Allied Health, University of Limerick, Limerick, Ireland; 6 School of Applied Psychology, University College Cork, Cork, Ireland

**Keywords:** Open Research, Open Educational Resource

## Abstract

**Background: **Open research involves actions at all stages of the research cycle to make the research process and outputs more transparent and accessible. Though a number of initiatives exist for researchers at PhD, post-doctoral and more senior levels, there remains a critical need for educational resources for research students at earlier career stages and across disciplines. The aim of the Principles and Practices of Open Research: Teaching, Research, Impact, and Learning (PaPOR TRaIL) project is to develop an open educational resource (OER) on the principles and practice of open research for undergraduate and master’s students.

**Methods:** In stage 1, interviews and surveys of students and supervisors are being conducted to explore students’ and supervisors’ knowledge, attitudes, and experiences of open research, in addition to needs and preferences for the content and delivery of the OER. Stage 2 involves development of the OER content and delivery, based on Stage 1 engagement and national and international guidance on best practice in conducting and teaching open research. In Stage 3, students and supervisors will evaluate the developed OER and provide feedback in terms of OER usability, learning experience and learning outcomes. This feedback will guide revisions and finalisation of the OER content, format and learning activities.

**Discussion:** The PaPOR TRaIL project will develop an evidence-based OER that provides a foundation in all aspects of open research theory & practice. Teaching undergraduate and master’s students open research will promote development of core research values and equip them with transferable competencies and skills, including how to conduct and use research in a trustworthy and ethical manner within and beyond academia. Enhancing teaching and learning of open research will promote better teaching and research outcomes that will benefit individuals, universities, and science more broadly.

## Introduction

Research practices that are open and transparent are essential to maximise the validity and impact of research ( Cox & Toomey, 2018). In addition to the conduct of research, open dissemination of research findings is crucial to ensure access to knowledge for both those who *produce* and those who *engage* with ** research. Open research is an umbrella term that incorporates a range of principles and practices that make research more transparent, reproducible and accessible to everyone in the society, leading to increased rigour, accountability, and collaboration ( Bezjak *et al.*, 2018.; Munafò *et al.*, 2017). Open research principles and practices are relevant at all stages of the research cycle, from study design to results dissemination. These practices ensure that the results of research are available to researchers and the general public, and also that the “hypotheses, materials, data, and procedures” that comprise research are accessible also ( Toelch & Ostwald, 2018, p.1). Open research is applicable to all disciplines, though it has been predominantly highlighted in the sciences and medicine, (e.g., Edmond & Tóth-Czifra, 2018; Peels & Bouter, 2018). As such, the term ‘open science’ was originally coined to reflect this focus, while the term ‘open research’ captures the same message and approaches and allows for greater inclusivity across disciplines. Further, though the principles of open research are consistent across disciplines, open research practices can be implemented differently across disciplines, thereby enhancing the transparency and robustness of research within and across disciplines ( Knöchelmann, 2019; Peels & Bouter, 2018; Wouters & Haak, 2017).

Benefits of open research have been noted for individual researchers, as well as advancing the more general goals of all research. For instance, it has been reported that more than 80% of university-level researchers agreed that open science could improve the quality of their research, and the majority reported positive attitudes towards open science ( Toelch & Ostwald, 2018). Benefits of open science cited by researchers include improved discoverability and the citation advantage of open access articles, as well as shorter publication embargo periods ( Allen & Mehler 2019; McKiernan *et al.*, 2016; Munafò *et al.*, 2017). In addition, engagement in open research practices provide a means by which to address and mitigate questionable research practices (QRPs; John *et al.*, 2012). Lack of transparency and QRPs can lead to increased mistrust in research findings ( Marcus & Oransky, 2014), research waste ( Chalmers & Glasziou, 2009; Macleod *et al.*, 2014), and can have potential consequences for the consumers of research, including for public and patient health and safety ( Herrera-Perez *et al.*, 2019; Prasad *et al.*, 2013). Engaging in open research practices, including making research data, analysis code, and research outputs openly accessible can ensure more transparency in research processes and address concerns about research reliability ( Open Science Collaboration, 2015). In addition, open research practices support the democratisation of scientific knowledge, and offers the wider community insights into the development and conduct of research and so enables a greater number of people to engage with research (including as consumers, beneficiaries and citizens) in an informed way ( Arza & Fressoli, 2017). 

Efforts to promote open research are not yet matched by uptake of open research practices among researchers ( Allen & Mehler, 2019; Baker, 2016). Lack of knowledge and training on open research is an important factor influencing application of open research in practice ( Working Group on Rewards under Open Science, 2017; Zečević *et al.*, 2020). In particular there is a critical need for training and education in open research principles and practices early in students research experience, during the “grassroots” stage ( Allen & Mehler, 2019; Button *et al.*, 2020, p.77; Strand & Brown, 2019). However open research educational resources are not typically aimed at undergraduate and master’s-level students, despite this serving as the foundations to everything that follows ( Button, 2018). In addition to a gap in accessible resources directed at undergraduate and master’s-level students, particularly in Ireland, there is also a gap in knowledge about engagement and uptake of resources by students. Exploring undergraduate and master’s-level students perceived knowledge, attitudes and experiences of open research, in addition to their needs and preferences for educational supports is essential to inform development and implementation of appropriate, relevant and meaningful educational resources ( Toelch & Ostwald, 2018). Similarly, engagement with the supervisors of undergraduate and master’s-level student research is critical to inform development of resources that are fit for purpose within the supervisor-student relationship. As such, the Principles and Practices in Open Research: Teaching, Research, Impact and Learning (PaPOR TraIL) project was established to develop a student and supervisor focused teaching and learning resource. By educating and engaging undergraduate and master’s-level students in open research principles and practices, the PaPOR TraIL project aims to provide a foundation in best scientific practice that benefits students, universities, and research as a whole.

### Aims

This project aims to develop an open educational resource founded upon the principles and practices of open research, in order to promote and facilitate the teaching and learning of open research for undergraduate and master's-level students in Ireland.

To inform the development of this resource, this study has three key objectives

1) To examine self-perceived knowledge, attitudes and experiences of open research among undergraduate and master’s-level students, and among research supervisors in Irish universities
2) To examine what students and supervisors deem to be useful in learning and practising open research
3) To pilot test the educational resource with students and supervisors and gain feedback to inform refinements and finalisation of the educational resource

### Design

This study uses a convergent mixed methods design, with qualitative and quantitative data collected concurrently. Two self-administered online surveys and interviewer-led, semi-structured online interviews and/or focus groups are being used to collect data from undergraduate and master’s-level students, and research supervisors, on their current self-perceived knowledge, attitudes and experiences of open research and on needs and preferences for teaching and learning resources. Data will be integrated via narrative synthesis, with quantitative and qualitative findings reported in separate sections within the same paper. The survey and interview topic guides are available as *Extended data* ( Matvienko-Sikar *et al.*, 2020).

### Setting

Data will be collected across Irish Higher Education Institutes. Student recruitment and data collection will take place in University College Cork (UCC). Data from research supervisors will also be collected in UCC, with requests for research data collection also sent to seven other Irish higher level institutions (University of Limerick, National University of Ireland Galway, Dublin City University, University College Dublin, Trinity College Dublin, Technological University Dublin, and Maynooth University). The data collection will be predominantly online, due to on-going COVID-19 restrictions.

### Participants and recruitment

Participants in this study are:

1) Students in UCC (aged 18 years and over) who are currently conducting or have conducted a research project as part of their undergraduate or master’s degree, or who have engaged in an extracurricular research project as part of a research team.
2) Academic or research staff members from UCC, University of Limerick, National University of Ireland Galway, Dublin City University, University College Dublin, Trinity College Dublin, Technological University Dublin, and Maynooth University (pending approval from higher education institutes), who have experience of supervising at least one undergraduate and/or master’s student research project to completion, within the last 2 years.

This study uses parallel sampling, with individual participants recruited from the same population pools (i.e., students and supervisors) participating in the qualitative and quantitative elements. Purposive sampling, utilising a maximum variation sampling approach is used to ensure a range of perspectives from students and supervisors across disciplines and at different educational levels. There are no exclusion criteria based on nationality or gender. There is also no exclusion criterion based on discipline of study because we are conscious that experiences, perceptions and support needs for open research may differ across disciplines and so an inclusive approach is adopted. Recruitment of undergraduate and master’s-level students in UCC is being conducted via a recruitment email sent to the entire student body. The email includes a link for the student survey and contact details to participate in a focus group or interview. It has been circulated to a body of approximately 18,000 students at the end of the 2019/2020 academic year and will be circulated again at the start of the 2020/2021 academic year.

Recruitment of research supervisors is via an email sent to all academic and research staff at the respective universities, where there is agreement to do so (e.g. from relevant offices, such as Teaching & Learning and Research Support Offices). The email includes a link for the supervisor survey and an invitation to participate in a focus group or interview, and the contact details of the research team should they wish to participate and/or have any further questions. Research supervisors will also be recruited via social media (e.g. Twitter) and through direct contact from research team members with academic and research staff in those universities. No sample size calculation was conducted for the survey, as this is a descriptive, exploratory study. For the qualitative data collection, it is estimated that interviews/focus groups will be conducted with approximately 20 staff and 30 student participants. Data collection and analysis will be conducted in an iterative manner and with an aim for data adequacy and a representative sample from different disciplines and educational level.

### Data collection

In order to facilitate integration of topic areas during analysis of the quantitative and qualitative data, the surveys and the focus group/interview topic guides have been developed to address similar topics, and are based on a review of open research literature ( Bezjak *et al.*, 2018; Munafò *et al.*, 2017; Toelch & Ostwald, 2018), and materials used to evaluate self-perceived open research knowledge, perceptions and experience among early career researchers ( Zečević *et al.*, 2020).

**Surveys.** Two self-administered online surveys will be used, with one survey for students and a second survey for research supervisors. Both surveys are comprised of four sections: 1) Demographics; 2) Knowledge; 3) Attitudes and 4) Preferences for learning resource types. The student survey demographic questions ask for student age, gender, year and discipline of study, and research experience; the supervisor survey demographics included university affiliation and experience of supervising undergraduate and master’s students. Student and supervisor self-perceived knowledge will be determined using 37 items that assessed 1) general perceived knowledge about open research, and 2) open research topics including data management, open access, knowledge dissemination, preregistration, and research integrity. Respondents will indicate their self-perceived knowledge by indicating agreement with statements about their open research knowledge using a five-point Likert scale from strongly agree to neutral and an ‘I don’t know’ option; items included “ *I have previously heard of the terms open research or open science*”, “ *Open Research applies across fields and disciplines*”. Attitudes about open research will be assessed using 26 items for all participants, and measured as for knowledge, using a 5-point Likert scale; items included “ *Open research can help me improve the quality of my research*”. Preference for learning content will be elicited by asking students to indicate usefulness of content areas, with 8 content areas presented, including 1) an introduction to open research, 2) pre-registration, 3) research and data management, 4) research integrity, 5) open reporting, 6) research reproducibility, 7) knowledge dissemination, or 8) ‘other’; items were measured using a five-point scale from not at all useful, to very useful. Preferences for content delivery will be elicited in the same manner, with 11 options including videos, templates, and examples of best practice; these are also measured using a Likert five-point scale from not at all useful, to very useful. A final question will ask participants to provide any additional information. The study surveys are included in Supplementary File 1.

**Focus groups and interviews.** One semi-structured topic guide each was developed for both student and supervisor focus groups and interviews. Focus groups were chosen as the initial primary data collection approach to generate group discussions among students and supervisors regarding self-perceived knowledge, attitudes and experiences of Open Research, as well as ideas about content and delivery of the open educational resource. Where focus groups cannot be facilitated, interviews are conducted. While interviews represent a distinct approach, they share similarities with focus groups in facilitating exploration of attitudes, beliefs and experiences in an in-depth manner. In the current study to date, interviews have been used as convening focus groups with students and supervisors have not been feasible given COVID-19 related restrictions and participants existing commitments. Similar to the student and supervisor surveys, the topic guide explores student and supervisor 1) self-perceived knowledge and attitudes of open research, 2) experience in applying open research practices, and 3) preferences and suggestions for appropriate learning resource types for students. Open questions to elicit opinions from participants are followed in the topic guides by additional prompts for each main question ( Robson, 2011). Please see Supplementary File 2 for the full topic guide.

### Analysis

**Survey data.** Descriptive statistics, including means and standard deviations of continuous variables, and frequencies of categorical variables, will be derived to examine supervisor and student levels of self-perceived knowledge and attitudes, as well as experiences and preferences for open research resources. Comparisons between groups (i.e. respondents from different disciplines) will be conducted, dependent on recruiting a final sample size that is appropriate for inferential statistics.

**Interview/focus group data.** The interview/focus group data will be analysed using inductive thematic analysis ( Braun & Clarke, 2006). Analysis of initial transcripts will be conducted in conjunction with data collection, and the data will be analysed iteratively to achieve data adequacy ( Braun & Clarke, 2019). Themes developed through inductive analysis of initial transcripts will serve as a framework for subsequent analyses, while also allowing scope for recognising and generating new themes. The inductive thematic analysis will be conducted using the qualitative analysis and data management software QSR NVivo V12. All data will be imported to NVivo and will be managed and coded in NVivo. The thematic analysis will be carried out in the six-phase, “iterative and recursive” way described by Braun and Clarke ( Braun & Clarke, 2006; Terry *et al.*, 2017). The six phases comprise: (1) familiarisation with the data, (2) code generation, (3) constructing themes, (4) reviewing themes, (5) defining and naming themes, (6) and writing up the final analysis ( Terry *et al.*, 2017). It is planned that coding will be conducted by one member of the research team. A second team member will check coding for approximately a quarter of the transcripts to ensure consistency credibility, accuracy and appropriateness of coding ( Nowell *et al.*, 2017). Differences in interpretation will be discussed to reach consensus; where resolution cannot be reached, a third coder will be consulted. Coding and themes developed will also be discussed within the broader research team. Individual interview and focus group data collected for each population (students and supervisors) will be analysed separately, with similarities and differences between the mode of qualitative data collection noted, and the type of data collection will be noted when referencing specific extracts in results. 

### Data integration to inform development of the open education resource

The qualitative and quantitative data will be integrated via narrative synthesis, using a contiguous approach, in which the qualitative and quantitative findings are reported in separate sections within the same report ( Fetters *et al.*, 2013). Findings related to self-perceived knowledge of open research, and attitudes towards and experiences of open research will be grouped, as will the findings on student and supervisor preferences for content and delivery options within the resource. Similarities and differences between staff and supervisors, and between qualitative and quantitative data collection will also be presented. These findings will be used to inform the content and delivery of the open educational resource on the principles and practices of open research. For instance, the findings will guide depth and breadth of the resource content in terms of which aspects are reported as most important to students and supervisors and which aspects students/supervisors report least self-perceived knowledge of and/or perceive students to require most support for. The findings will also inform the delivery format based on student and supervisor preference.

### Pilot testing

The PaPOR TRaIL open educational resource will be pilot-tested and evaluated by students and supervisors once developed. Students and supervisors will engage with the open educational resource and work through the content sections at their own pace and will then be invited to provide informal feedback. This feedback will take the form of an audio recorded discussion with a member of the research team about their experience of using the module in terms of module usability, learning experience and learning outcomes (including awareness, understanding, self-perceived knowledge and practices of open research). Feedback from all students and supervisors who pilot test the open educational resource will be transcribed and narratively summarised. The aggregated feedback will then be used to guide any revisions needed to content, format and learning activities in order to finalise the open educational resource.

### Research ethics approval and ethical considerations

Ethical approval for this study was granted by the Social Research Ethics Committee of University College Cork on 12/05/2020, Log 2020-080. Amended ethical approval to expand inclusion criteria for the research supervisor participant sample was granted by the same body on 19/08/2020, Log 2020-080A1. All students and staff who participate in the quantitative and/or qualitative data collection are provided with full study information and provide informed consent prior to data collection (a model consent form is available as *Extended data* ( Matvienko-Sikar *et al.*, 2020)). Participant confidentiality will be maintained throughout the study. For instance, data collected using online surveys are completed anonymously and no identifying information is collected, ensuring that there is no means of tracing the survey responses to corresponding participants. Participant details required for conducting focus groups and interviews are stored separately to qualitative data collection and are deleted following data collection. Focus group/interview transcripts will be anonymised using participant codes to disguise the identity of participants, thus protecting their privacy. All transcripts will therefore be stored in the anonymised formats. Only project members will have access to study data, which will be collected and processed in line with the six principles of the European General Data Protection Regulations (GDPR), the Irish Data Protection Act 2018 and the UCC Data Protection policy.

### Dissemination

A knowledge exchange and dissemination event will be held at the end of the project to share information about the developed open educational resource. This event will include students and research supervisors from Irish universities, as well appropriate representatives from research and teaching and learning offices within universities. The findings of the qualitative and quantitative data collection, and the overall development of the educational resource, will be reported in open-access peer-reviewed publications, and will be presented at conferences. The aggregated, anonymised, FAIRified study data will be made available upon publication of research articles via the Open Science Framework repository. This will include the anonymised quantitative datafile, the analysis plan, and any code used to analyse data. The qualitative data from focus groups/interviews will be made available in an aggregated and anonymous way, in the form of coding queries generated using QSR NVivo qualitative analysis and data management software.

In addition, social media (e.g. Twitter) will be utilised to engage with the open research, and teaching and learning communities online, to disseminate information about the project and educational resource. Visual approaches to dissemination, such as infographics and short videos, will be utilised to maximise dissemination on these platforms. Finally, the educational resource developed will be an open educational resource and, as such, will be made openly available to all higher education institutes.

### Study status

Data collection for this study has commenced and is on-going. Analysis of data has not yet commenced.

## Discussion

Open research principles and practices are crucial to improving the reliability of research and to guiding and supporting responsible research cultures. Introducing open research to university students at undergraduate and master’s-level will improve the capacity of students to understand the importance of transparent, accountable research practices at every stage of the research process and will support the development of responsible research practices and more informed engagement with research and knowledge generation. Creating an open educational resource guided by student and research supervisors needs and preferences supports the development of a relevant and meaningful educational resource that facilitates student use and engagement. The findings of this study, and development of the open research educational resource will contribute to informing and supporting the teaching and learning of open research at a critical juncture in a students research journey.

## Data availability

### Underlying data

There are no underlying data associated with this project.

### Extended data

Open Science Framework: Principles and Practices of Open research: Teaching, Research, Impact, and Learning (PaPOR TRaIL). https://doi.org/10.17605/OSF.IO/SJF32 ( Matvienko-Sikar *et al.*, 2020).

This project contains the following extended data:

- Supplemental-file-1-surveys.pdf
- Supplemental-file-2-Interview-Topic-Guides.pdf
- Supplemental-file-3-Consent-forms.pdf
- Supplemental-file-4-Information-sheets.pdf

Data are available under the terms of the Creative Commons Attribution 4.0 International license  (CC-BY 4.0).
