# Supplementary material for: Developing an open educational resource for open research: Protocol for the PaPOR TRAIL project
Source: HRB Open Res. 2020 Nov 12;3:84. [Version 1] doi: 10.12688/hrbopenres.13171.1 (PMC7737705; doi:10.12688/hrbopenres.13171.1)
Supplement: Peer review history

# Peer review: Reviewer response for version 1

**DOI:** 10.21956/hrbopenres.14296.r28350

**Published:** 2020-12-14

**Authors:** Ljiljana Tasić

**Affiliations:** 1 Faculty of Pharmacy, Department of Social pharmacy and Pharmaceutical legislation, University of Belgrade, Belgrade, Serbia

This manuscript is well written with core details of the study that are included in this protocol of the Project titled: Principles and Practices of Open Research: Teaching, Research, Impact, and Learning (PaPOR TRaIL).

 The Open Educational Resource (OER) project founded upon the principles and practices of open research, in order to promote and facilitate the teaching and learning of open research for undergraduate and master's-level students in Ireland; from the protocol and expected results, the impact and value of Project should be recognized not only at national (Ireland) but at international level, as well.

 This PaPOR TRIAL Project with Protocol presented in manuscript will be resulted with supportive module of integrative course during the complex process of the teaching and learning of researchers/scientist beginner at a critical juncture in their career journey.

 I suggest to authors to consider of academic and research environment as set of social and cultural context in which research occurs, as important for the student’s research journey. Some interesting and valuable detail you can find in MacLeod *et al* ^1^.

 My reflection, during the reading the manuscript, is mainly from the biomedical and health science perspective, were the open science principles and practice promulgated research capacity, recognizable from number of publishing articles, books etc. The principles of open research are consistent across disciplines, and open research practices can be implemented differently across disciplines. During the Pilot testing, that is the third step in study protocol, should be useful to collect and analyzed the students and supervisors feedback from across disciplines to enhancing the transparency and robustness of research.

 All over, above comments, consider as open discussion with peers.

 I'm really happy to read this a good manuscript and supportive to education society and academia, to improve open research practice with new education resources, and looking forward to assessing the OER, to read further publication and follow up the project in the future and expected the great impact.

Is the study design appropriate for the research question?

Yes

Is the rationale for, and objectives of, the study clearly described?

Yes

Are sufficient details of the methods provided to allow replication by others?

Yes

Are the datasets clearly presented in a useable and accessible format?

Yes

Reviewer Expertise:

Health system, Education, Pharmacy Practice research, Rational medicine use

I confirm that I have read this submission and believe that I have an appropriate level of expertise to confirm that it is of an acceptable scientific standard.

## References

[1] : Biomedical research: increasing value, reducing waste. *The Lancet*.2014;383(9912) : 10.1016/S0140-6736(13)62329-6 101-104 10.1016/S0140-6736(13)62329-6 24411643

# Peer review: Reviewer response for version 1

**DOI:** 10.21956/hrbopenres.14296.r28347

**Published:** 2020-12-02

**Authors:** Sam Parsons

**Affiliations:** 1 Department of Experimental Psychology, New Radcliffe House, Radcliffe Observatory Quarter, University of Oxford, Oxford, UK

I was very excited to read this manuscript. The authors describe the protocol for the PaPOR TRaIL (Principles and Practices of Open Research: Teaching, Research, Impact, and Learning) project, which takes a mixed methods approach to explore the open research content most applicable to undergraduate and masters students. I was particularly impressed that one of the key outcomes is the development of an open educational resource that the wider community can benefit from.

 During my reading of the manuscript, and writing of this review, I have kept in mind conversations I have had with educators of open research as part of the FORRT project (Framework for Open and Reproducible Research Training). FORRT and PaPOR TRaIL have overlapping goals, and I wanted to begin this review by commenting on how important I believe open educational resources are, and therefore how encouraged I am by the PaPOR TRaIL project. Outside of this review, it would be good to explore the overlaps between projects and how they might benefit one and another. While I don’t see this as a conflict of interest, I think it is worth noting up front.

 One aspect of the paper that I was not entirely clear on until later on in the paper was the target audience of the educational resource. Perhaps I came with my own preconceptions of what the project and the OER would entail. But, initially I thought that the OER would be a general resource for undergraduates and masters students to impart knowledge of Open Research – for instance to be delivered as part of teaching rather than as part of a research project. Reading further, and reflecting on the participants, my reading is that the resource would be aimed at students and supervisors currently working on research projects. I wonder if this would miss an ideal time to learn about Open Research – before conducting research. More importantly, if this is the case, then I wonder if it could be made clearer earlier on that this will be the target audience for the OER? If, however, the OER is more general – for instance, so that instructors could use it as part of a methods course to teach open research practices – then I wonder if there are some stakeholders missing from the discussions. For instance, if instructors are expected to deliver the OER, then perspectives on how it could be incorporated into other courses or delivered in such a way as to not overly burden them would be valuable additions to the project. It may also be valuable to seek wider input from other instructors during the pilot phase of the OER (this is something the FORRT community could also facilitate).

 Overall, the manuscript does an excellent job of describing the details of the project. I particularly like the use of Open Research as being more inclusive than Open Science. I only have a few comments on other details that the reader might benefit from.

1. The lack of resources aimed at undergraduates and masters students is important to consider. The authors mention that this is particularly the case in Ireland. Are there reports of institutions that cover open research educational resources? Or could the authors clarify that this is from their collective assessments of the landscape of their institutions?
2. I agree that open research supports the democratisation of research knowledge, and has the power to increase the equity of the entire research endeavour (though this will depend on the degree to which this is the intention versus an outcome we merely hope for – there have been several discussions about how the Open Research community and many prominent members reflect the existing lack of diversity and there is much to do in this area). With respect to this, I wonder if the authors could reflect and comment here or when disseminating the OER how applicable it is to other countries, contexts, and communities, as well as a range of research areas. It may also be worth reflecting on who is represented in the topics and resources covered in the OER to maximise representation. This is not a critical point of what the authors have included and this may already be an important consideration, but it is worth raising as materials are developed.
3. Is there a proposed date for the end of data collection?
4. I wonder if joint discussions with supervisors and their students when piloting the OER would be useful?

I have two other comments about the project more broadly that may not apply to revisions of this manuscript directly.

1. This would likely be outside of the scope of the project. Yet, I cant help think the perfect outcome to assess would be the students research project write-up. An assessment of the open research practices included in the studies themselves would provide information on how the OER has influenced actual practices, as well as understanding of the practices.
2. This is perhaps more of a thought for the final analyses. I wonder if and how the authors could account for varying prior knowledge of open research practices. In so far as greater knowledge about these practices should help students and supervisors pinpoint the practices they need to know more about (both for the students enacting them and the supervisors supporting/teaching them). Whereas, low knowledge of these practices may lead to little potential for reflection on how their research could have improved with those practices.

The PaPOR TRaIL promises to make several important pedagogical contributions. These very minor points aside (some of which are more applicable to the future work on this project rather than any issues I have with the manuscript), this manuscript is very well written and extremely detailed. All of the core details of the study are included in this protocol and the project is set up to be rigorous and informative. The aims are clearly stated and the methods match them nicely. I, and I suspect many others, are looking forward to accessing the OER and reading more about the project in the future.

 In the interest of openness I always sign my reviews,

 Sam Parsons

Is the study design appropriate for the research question?

Yes

Is the rationale for, and objectives of, the study clearly described?

Yes

Are sufficient details of the methods provided to allow replication by others?

Yes

Are the datasets clearly presented in a useable and accessible format?

Yes

Reviewer Expertise:

Open research, affective cognition, psychometrics.

I confirm that I have read this submission and believe that I have an appropriate level of expertise to confirm that it is of an acceptable scientific standard.
